# Trends in the prescription of benzodiazepines for the elderly in Korea

**DOI:** 10.1186/s12888-017-1467-z

**Published:** 2017-08-22

**Authors:** Soo-Hee Hwang, Seungjin Han, Hyojung Choi, Choonseon Park, Sun Min Kim, Tae Hyun Kim

**Affiliations:** 1 Health Insurance Review and Assessment Service (HIRA), Wonju, Republic of Korea; 2 Department of Hospital Administration, Graduate School of Public Health, Institute of Health Services Research, Yonsei University, 50-1 Yonsei-ro, Seodaemun-gu, 03711 Seoul, Republic of Korea

**Keywords:** Benzodiazepines, Elderly, Inappropriate drugs, Prescribing quality indicators

## Abstract

**Background:**

This study examined trends in the prescription of benzodiazepines for the elderly (age over 65 years) in Korea, a country with a higher level of spending on pharmaceuticals compared to that in other Organization for Economic Cooperation and Development (OECD) countries, and identified factors related to the inappropriate use of such drugs.

**Methods:**

We used the National Health Insurance Claims Data (NHICD) for the period 2009–2013, including all reimbursed drug-prescribing information. Following the OECD’s prescribing quality indicators (PQIs), we looked at the prevalence, quantities, durations, and inappropriate (long-term or high-quantity) use of benzodiazepines, some of the most widely prescribed, but potentially inappropriate, drugs for the elderly. We also performed multivariate logistic regression analyses to identify factors related to the inappropriate use of these drugs.

**Results:**

The annual prevalence of benzodiazepine prescribing for elderly subjects decreased slightly over time but remained high (37.9% in 2009 and 35.1% in 2013). There were also small decreases in the inappropriate long-term use of benzodiazepines over the five years, with a 0.6 decrease in the Defined Daily Dose and a 4.1 per 1,000 decreases in elderly user-days. The proportion of subjects using long-acting benzodiazepines also fell from 263.6 to 220.4 per 1,000 elderly patients. The regression analyses found that the inappropriate long-term use of benzodiazepines in the elderly was significantly related to the patients visiting several institutions and physicians prescribing more than 30 days’ worth of medication.

**Conclusions:**

The prevalence of prescribing potentially inappropriate drugs, such as benzodiazepines, remains high in Korea. Policy efforts, such as a periodic assessment of prescribing, restricting prescribing days, and more practical guidelines, are needed to improve the quality of prescribing.

## Background

As some of the most widely prescribed drugs, benzodiazepines and related drugs are commonly used among the elderly for the treatment of anxiety, insomnia, muscle spasm, and epilepsy, despite the well-known risks for older people [1–4]. According to the recently updated Beers criteria, it is strongly recommended that benzodiazepines be avoided for the treatment of insomnia, agitation, or delirium in older people, because such patients have an increased sensitivity to the drugs and slower metabolism [5]. However, older people are still the largest consumers of benzodiazepines [1, 6, 7], and this may be associated with poor health outcomes, such as falls, fractures, cognitive impairment, and dependence [4, 8].

Recently, the Health Care Quality Indicator (HCQI) project of the Organization for Economic Cooperation and Development (OECD) introduced prescribing quality indicators (PQIs) for primary health care. Among these, some medication safety indicators are used as prescribing indicators to measure the potentially inappropriate use of benzodiazepines in the elderly, because this therapeutic area has stood out as one of the most concerning issues in many countries [9].

Korea has higher levels of spending on pharmaceuticals compared to other OECD countries, with pharmaceuticals accounting for approximately 20.6% of the total expenditure on health insurance in 2013. The country has also been rapidly moving toward an aged society, with the elderly population rising sharply [10]. A systematic intervention and monitoring system for managing the quality of prescriptions in older people at the government level is needed in Korea. [7] A recent study showed that benzodiazepines were one of the most common medications prescribed inappropriately to aged people in Korea, based on the American Geriatric Society updated Beers criteria list. [11] The Korean government started enforcing a policy regulating the number of prescribing days for sedatives and hypnotics (< 30 days per prescription) in 2010 [7]. However, so far, there have been no federal policies/strategies to manage the full spectrum of benzodiazepines.

The aims of this study were to investigate the prescribing patterns and prevalence of the inappropriate use of benzodiazepines for older people in Korea by applying the newly developed PQIs of the OECD HCQI project to identify the scale of potentially problematic benzodiazepine prescribing. We also examined the potential risk factors related to the inappropriate use of benzodiazepines to discuss and suggest changes to the existing policy.

## Methods

### Data source

The National Health Insurance Claims Data (NHICD) submitted to the Health Insurance Review & Assessment Service (HIRA) for the period from 2009 to 2013 was used for this study. The study period included 2010, when the new regulation went into effect limiting the prescription days of some classes of sedatives and hypnotics. The HIRA is in charge of claims review and quality improvement under Korea’s single-payer health insurance system, and the HIRA’s database broadly represents the Korean population in terms of sex, age, and geography [12]. The database contains de-identified information on the patients’ age, gender, type of insurance (National Health Insurance or Medical Aid, a public assistance program for low-income households that assists with self-help by providing medical services), diagnoses, procedures and operations, type of medical services, institution(s) visited, visit dates, prescriber’s identification number and prescribed and dispensed medications [13]. The database also contains detailed prescription and dispensing information, including Korean drug codes, brand names, quantities, and durations of prescribing and dispensing. For a more accurate analysis, we used the dispensing data.

### Study subjects

The study population consisted of outpatients aged over 65 years as of July 1st each year who were prescribed benzodiazepines and related drugs at least once during the study period. The World Health Organization (WHO) Anatomical-Therapeutic-Chemical (ATC) coding and categorization system for drug data coding was used to classify medications. Benzodiazepines and related drugs include ATC codes N05BA (anxiolytics containing benzodiazepine derivatives), N05CD (hypnotics containing benzodiazepine derivatives), N03AE01 (clonazepam, antiepileptics), and N05CF (hypnotics containing benzodiazepine-related drugs).

Patients who were prescribed benzodiazepines that were not assigned a Defined Daily Dose (DDD) were excluded from this study (clotiazepam, etizolam, pinazepam, etc., approximately 10.7% of the patients). We also excluded subjects prescribed benzodiazepines for conscious sedation endoscopy (approximately 3.7%).

### Measures of inappropriate prescribing

The inappropriate use of benzodiazepines for the elderly was measured using the newly proposed PQIs in the primary care of the OECD’s HCQI project. The PQIs for the inappropriate prescribing of benzodiazepines included the long-term use of benzodiazepines (patients who were prescribed benzodiazepines over 365 DDDs/year, 567.5 DDDs/year, 365 days/year, or 547.5 days/year) or who received at one or more prescription of long-acting benzodiazepines [9]. Of these five indicators, we only considered the long-term use of benzodiazepines as the use over 365 DDDs/year, 365 days/year, and the use of long-acting benzodiazepines because the thresholds of 567.5 DDDs/year and 547.5 days/year were too high and had a very low prevalence.

### Statistical analysis

We focused on the usage patterns and safety of benzodiazepine prescriptions for older people. The prevalence of benzodiazepine prescriptions in the elderly population and the mean prescription quantities (DDD) and durations (user-days) were calculated with respect to the patient age, sex, and type of insurance. The overall volume of benzodiazepine prescription was presented as the DDDs and user-days and per 1000 elderly population per day. The inappropriate use of benzodiazepines was presented as the rate per 1000 elderly population each year. Finally, we carried out multivariate logistic regression analyses on the benzodiazepine users in 2013 to investigate the factor potentially related to inappropriate use. The analyses were adjusted for each demographic characteristic, the number of institutions utilized, and the main disease cited as the indication for benzodiazepine prescription. Adjusted odds ratios (ORs) with 95% confidence intervals (CIs) were calculated.

Data extraction and statistical analyses were conducted with Statistical Analysis Software (SAS) Enterprise Guide Version 4.3 (SAS Institute, Inc., Cary, NC). Denominator information on the Korean population was downloaded from Statistics Korea (http://kosis.kr/statisticsList/statisticsList_01List.jsp?vwcd=MT_ZTITLE&parentId=A#SubCont).

## Results

### Prevalence and patterns of benzodiazepine prescriptions

The study population was about 5.3 million elderly in 2009 and 6.3 million elderly in 2013, and the number of patients prescribed benzodiazepines at least once was about 2.0 and 2.2 million elderly, respectively. From 2009 to 2013, the prevalence of patients prescribed benzodiazepines slightly decreased from 37.9% to 35.1% (−2.8%), with the smallest decline in the 80–84 age group (−0.8%). However, in the Medical Aid group, the prevalence of benzodiazepine prescriptions rose from 12.7% to 13.5% (+0.8%). Females were about 10% more likely to be prescribed benzodiazepines compared to males (Table 1).

**Table 1:** Prevalence of benzodiazepine prescriptions from 2009 to 2013

		2009	2010	2011	2012	2013	Difference 2009–2013
		(%)					
Total		37.9	37.2	36.5	36.1	35.1	−2.8
Age group (year)	65–69	36.4	35.9	34.5	34.1	31.6	−4.8
	70–74	39.8	39.1	38.2	37	37.2	−2.6
	75–79	40.6	39.6	39.7	39.7	38.7	−1.9
	80–84	38.1	37.8	37.6	37.6	37.3	−0.8
	85+	30.3	28.4	28.7	28.8	28.8	−1.5
Sex	Male	31.1	30.7	30.4	30.1	29.3	−1.8
	Female	42.6	41.7	40.8	40.2	39.2	−3.4
Type of Insurance	NHI	50.2	48.1	46.1	44.1	41.8	−8.4
	Medical Aid	12.7	12.3	12.7	13	13.5	0.8

*NHI* National Health Insurance

**Fig. 1:**
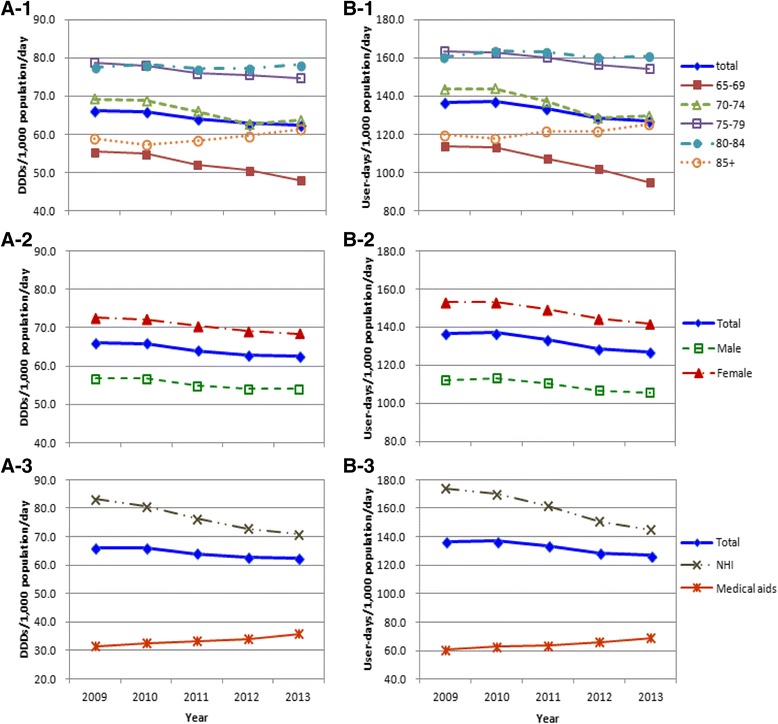
The patterns of benzodiazepine prescriptions in terms of quantities (A) and durations (B) by age (1), sex (2), and type of insurance (3). NHI = National Health Insurance

The overall volume of benzodiazepine prescriptions among the elderly in 2009 was 66.1 DDDs/1000 population/day and 136.6 days/1000 population/day, but it steadily declined over the five-year period by 3.7 DDDs and 9.7 days in terms of the quantity and duration, respectively. However, the patterns of benzodiazepine prescriptions in the older (80–84- and over 85-year-olds) and Medical Aid groups remained nearly unchanged or increased (Fig. 1).

### Inappropriate long-term use of benzodiazepines

The proportion of patients prescribed more than 365 DDD/year of benzodiazepines steadily decreased from 12.2/1000 elderly in 2009 to 11.4/1000 elderly in 2011, but the rate remained unchanged during the next two years. For the Medical Aid group, however, the proportion of long-term use of benzodiazepines increased by 0.4/1000 elderly over the five years.

In terms of the duration, the proportion of patients prescribed more than 365 days/year also decreased from 42.6/1000 elderly in 2009 to 38.5/1000 elderly in 2013. The extent of the reduction of the inappropriate use over the study period decreased with age, and the inappropriate use increased in the oldest age group (85+). In the Medical Aid group, the value also increased by 0.7/1000 elderly over the five years (Table 2).

**Table 2:** Inappropriate long-term use of benzodiazepines prescriptions from 2009 to 2013

			2009	2010	2011	2012	2013	Difference 2009–2013
			(numbers per 1000 elderly population)					
DDD	Total		12.2	12	11.4	11.6	11.6	−0.6
	Age group (year)	65–69	10.1	9.9	9.4	9.6	9.3	−0.8
		70–74	12.9	12.7	11.9	11.6	12	−0.9
		75–79	14.7	14.5	13.6	14	13.9	−0.8
		80–84	14.7	14.3	13.5	14.1	14.1	−0.6
		85+	10.2	9.5	9.4	9.9	10.1	−0.1
	Sex	Male	11.3	11.2	10.5	10.6	10.8	−0.5
		Female	12.9	12.6	12	12.2	12.2	−0.7
	Type of Insurance	NHI	14.9	14.5	13.3	13.1	13	−1.9
		Medical Aid	6.8	6.5	6.7	7.1	7.2	0.4
User-days	Total		42.6	43	41.3	39.2	38.5	−4.1
	Age group (year)	65–69	34.1	34.2	31.9	29.7	27.7	−6.4
		70–74	45.2	45.4	42.5	39.1	39.3	−5.9
		75–79	52.5	52.9	50.9	49.3	48.5	−4.0
		80–84	51.6	53	52.3	50.2	50.3	−1.3
		85+	35.9	35.8	36.5	36.6	37.1	1.2
	Sex	Male	35.2	36	34.8	32.8	32.6	−2.6
		Female	47.5	47.8	45.7	43.7	42.8	−4.7
	Type of Insurance	NHI	53.5	53.3	49.7	45.5	44.1	−9.4
		Medical Aid	20.2	19.7	20.3	20.9	20.9	0.7

*NHI* National Health Insurance

When inappropriate use was assessed based on the prescribing of long-acting benzodiazepines, another measure of inappropriateness, there were 264 individuals per 1000 elderly people who were prescribed long-acting benzodiazepines in 2009, showing a decreasing pattern over the five years (−43.2/1000 elderly). Similar to the other measures, there was little improvement in the Medical Aid group (Table 3).

**Table 3:** Inappropriate use of long-acting benzodiazepines from 2009 to 2013

		2009	2010	2011	2012	2013	Difference 2009–2013
		(numbers per 1000 elderly population)					
Total		263.6	252.9	242.1	233.6	220.4	−43.2
Age group (year)	65–69	255.8	246.5	229.7	219.8	196.5	−59.4
	70–74	281.7	272.2	259.3	246.1	240.7	−41.1
	75–79	282.8	270.8	266.8	262	249.7	−33.1
	80–84	254.7	246.4	240.9	236.7	227.9	−26.8
	85+	187.2	169.4	165.5	161.5	155.5	−31.6
Sex	Male	210.2	202.8	194.8	187.3	176.6	−33.6
	Female	299.9	287.3	274.9	266.1	251.6	−48.3
Type of Insurance	NHI	352.9	328.9	307.6	287	264.4	−88.5
	Medical Aid	80.7	80.3	79.1	79.5	79.7	−1.0

*NHI* National Health Insurance

### Factors potentially related to inappropriate long-term use

Males and the Medical Aid group were significantly more likely to have inappropriate benzodiazepine use, but the effect of age produced contradictory results in the DDDs and user-days model. In addition, we found that inappropriate benzodiazepine use was strongly associated with the utilization of multiple health care providers throughout the year, the presence of at least one benzodiazepine prescription for 30 days or more, and the receipt of at least one benzodiazepine prescription for a psychiatric disorder (Table 4).

**Table 4:** Adjusted odds ratios with 95% CIs of inappropriate long-term benzodiazepines use, 2013

Characteristics		DDDs			User-days		
		Adjusted Odds ratio (95% CIs)		*p* value	Adjusted Odds ratio (95% CIs)		*p* value
Age group (per 10 years)		0.995	(0.994, 0.996)	<.001	1.006	(1.005, 1.007)	<.001
Sex	Male	1.366	(1.345, 1.389)	<.001	1.137	(1.125, 1.148)	<.001
	Female	1.000	Ref.		1.000	Ref.	
Type of Insurance	NHI	1.000	Ref.		1.000	Ref.	
	Medical Aid	1.398	(1.367, 1.431)	<.001	1.178	(1.160, 1.195)	<.001
No. of utilization of institutions	1	1.000	Ref.		1.000	Ref.	
	2	1.555	(1.526, 1.584)	<.001	1.54	(1.523, 1.557)	<.001
	3	1.888	(1.843, 1.933)	<.001	1.888	(1.858, 1.918)	<.001
	4	2.239	(2.165, 2.315)	<.001	2.265	(2.210, 2.322)	<.001
	5+	2.966	(2.850, 3.087)	<.001	3.199	(3.095, 3.306)	<.001
At least one prescription w/ + 30 days	No	1.000	Ref.		1.000	Ref.	
	Yes	3.368	(3.314, 3.423)	<.001	8.237	(8.156, 8.318)	<.001
At least one prescription for psychiatric disorders	No	1.000	Ref.		1.000	Ref.	
	Yes	7.050	(6.925, 7.177)	<.001	2.946	(2.917, 2.976)	<.001

*NHI* National Health Insurance, *CI* Confidence Intervals

## Discussion

### Prevalence and patterns of benzodiazepine prescriptions

The results of the study indicate that there was a high prevalence and a slightly decreasing trend in the prescribing of benzodiazepines and related drugs in the elderly during the study period. The prevalence of benzodiazepine prescriptions among the older population in our study was higher than in other countries (8.7–31.9%), although there were some differences in the ages among the studies (over 65 years, 65–80 years, and over 60 years) [1, 14–16]. Compared with previous reports on the use of benzodiazepines in older people in Korea, the prevalence rate was lower than the 50.3% observed among elderly outpatients in 2005 and 2006 and the 46.0% shown in the 5% sampling data collected for all settings between 2007 and 2011 [7, 17]. These differences may be mainly due to the inclusion and exclusion criteria, because we excluded the ~10% of patients who received benzodiazepine prescriptions that were not assigned an ATC-DDD for international comparison. Regardless of the differences between studies, benzodiazepines are still widely prescribed for the elderly in Korea. Moreover, the total volume of benzodiazepines prescribed has recently increased for the older subjects and patients receiving Medical Aid.

Our results suggest that the patterns of benzodiazepine use by the elderly in Korea are concerning, because these drugs are still very commonly prescribed. Although they tend to be low-dose prescriptions, the drugs are prescribed for the long-term, and their use is increasing for more vulnerable patients.

### Inappropriate use of benzodiazepines

In this study, we considered 365 DDDs/yr. and 365 days/yr. the cut-off point to define inappropriate use. Even with these very generous criteria, more than 11 per 1000 elderly population in terms of the DDDs and more than 38 per 1000 elderly population in terms of user-days were estimated to have been inappropriately prescribed benzodiazepines. This accounted for 3.3% and 11.0% of the elderly benzodiazepine users in terms of the DDDs and user-days in 2013, which was much higher than the rate indicated in a recent German report (4.8 per 1000 elderly based on the DDDs) [18]. However, according to the benzodiazepine use in the North Eastern Health Board region of the Republic of Ireland in 2002, 9.7% of benzodiazepine patients in the General Medical Service (which supplies all medicine to the socially-disadvantaged and people ≧ 70 years) had prescriptions every month during a year [19]. Because direct comparisons of different studies were hampered by the different definitions of long-term or chronic use (e.g., repeated sporadic use, prescriptions 100–365 days, or 100–365 DDDs and over per year, etc.), the units of measure, and the study populations, we are unable to determine whether these levels were higher or lower than those of other countries [1, 18–25].

However, the use of long-acting benzodiazepines in the elderly population is concerning, because the Beers guideline strongly advises that they not be used by older people except for specific indications such as seizure disorders, benzodiazepine withdrawal, ethanol withdrawal, severe generalized anxiety disorder, and periprocedural anesthesia. Despite the trend toward a slow decrease in inappropriate use, over one-fifth of the elderly population and nearly two-thirds of elderly benzodiazepine users still received at least one prescription for a long-acting benzodiazepine. This was almost 7 times higher than the results in the German study (242.1 in Korea vs. 33 per 1000 elderly at 2011) and 2.6 times higher than that of American benzodiazepine users aged 65 to 80 (69.5% in Korea in 2009 vs. 23.8% in the United States in 2008) [1, 18]. This is consistent with a study result that showed that long-acting benzodiazepines accounted for 53.7% of the prescriptions of benzodiazepines in Korean elderly outpatients [17].

This result indicates that the prescribing of long-acting benzodiazepine in this population was at a high level, which is against the advised guidelines, and a considerable number of elderly patients have been exposed to a high risk of sedative effects and psychomotor impairment.

### Factors potentially related to inappropriate long-term use

All of the factors we considered may contribute to the inappropriate long-term benzodiazepine use in the elderly. In particular, the elderly patients and physicians’ behaviors (e.g., the same patient visiting multiple institutions within a year and physicians prescribing at least one benzodiazepine prescription for ≧ 30 days) as well as the patients’ demographic and clinical characteristics, might have affected the inappropriate long-term use of benzodiazepines.

There is no systematic intervention and monitoring system that functions as a gatekeeper to control medical use across multiple health care institutions, and regulations for limiting prescription days at a single visit have been applied only for some hypnotics/sedatives and triazolam in Korea. [7] In this situation, physicians may have difficulty raising the issue of dependence and applying benzodiazepine withdrawal programs, because patients may transfer to another practitioner who will meet their demands [19].

In addition, the receipt of at least one benzodiazepine prescription for psychiatric disorders (e.g., anxiety, depression, insomnia, and ordinary life stresses) might be another key factor leading to inappropriate use among elderly patients. Benzodiazepines are initially efficacious for these conditions, but there are withdrawal symptoms, especially in elderly patients [26]. There is a lack of information available on the long-term effectiveness of benzodiazepines, and there are increasing concerns about the high risk of adverse events, but prescribers and patients do not always accept such information [20, 27]. Unfortunately, well-established guidance on good benzodiazepine prescribing or managing strategies for patients with persistent benzodiazepine misuse (e.g., an algorithm for a benzodiazepine reduction program, education, patient information leaflets) does not exist in Korea, mainly due to the low priority placed on and the low level of interest in the quality of prescriptions in primary care.

This discordance between prescribing guidelines and the practice environment and the shortage of guidelines and information on the safe use of benzodiazepines have been postulated as potential explanations for the widespread use of benzodiazepines and the lack of improvement in their inappropriate use.

### Implications for policy

The results of the present study indicate that the substantial inappropriate use of benzodiazepines and related drugs may be related to the absence of a “gatekeeper” or family doctor in Korea and the absence of official guidance for benzodiazepine prescribing. Because the earlier efforts of the government to limit the prescribing days for some classes of benzodiazepines led to some improvement of benzodiazepine prescription, it is clear that additional policies on the safe use of benzodiazepines and related drugs may be helpful to tackle this problem [7]. First, concurrent drug utilization review (DUR) services, which enroll all people in the national health insurance program and provide safety information in real-time at the stage of prescribing and dispensing [28], may need to add review rules to identify potentially inappropriate benzodiazepine prescriptions in the elderly. Unlike US pharmacy benefit managers, the Korean DUR services mainly focus on drug–drug interactions, ingredient duplications, drug–age conflicts, and drug use during pregnancy, and they have been limited to certain ingredients [29]. Furthermore, there has been no mechanism to ensure the periodic assessment of the various aspects of prescribing, such as an inappropriate treatment duration or over-utilization. Therefore, providing information on the cumulative quantity of potentially inappropriate medications during or after patient treatment will be necessary.

Second, regulations for restricting prescriptions to a 30-day supply at a visit should be considered for all kinds of benzodiazepines. Our analysis shows that the patterns of benzodiazepine use in the Korean elderly were low-dose/long-term, and receiving at least one prescription lasting ≧ 30 days for benzodiazepines was positively associated with inappropriate use. However, limiting the prescription days for drugs with a high risk of inappropriate use, such as triazolam, has had a little effect on the total quantity of benzodiazepines prescribed [7], so it is necessary to expand the items considered for restricted prescription days for the elderly.

Third, national efforts to make the guidelines more in tune with Korean practice and the widespread dissemination of information on the low effectiveness and serious risks of long-term benzodiazepine use will be needed. Although researchers developed a list of potentially inappropriate drugs for the elderly population of Korea in 2010, it has not been widely accepted [11, 30]. Patients and physicians’ perceptions and behaviors are key factors that determine the use and misuse of benzodiazepines, and thus, more practical guidelines, education, and campaigns should be considered, especially for elderly patients suffering from psychiatric disorders.

Although there might be some concerns about the unexpected results of stricter enforcement of regulations [7, 20], the benefits of improving benzodiazepine use and safety in the elderly will outweigh any drawbacks.

### Limitations

This study is not without several limitations. Since no detailed information about the prescribing physician was recorded in the database, it was not possible to identify physician-related factors, such as the prescribers’ views on experiences with benzodiazepines. Second, we analyzed benzodiazepine use in the elderly based on filled-prescription and dispensing data, which may not necessarily reflect actual use. Thus, our analysis might have led to an overestimation of benzodiazepine use and inappropriate use. However, because of their properties and association with dependence, it is unlikely that these drugs went unused, especially in long-term users, so we assumed that the prescription and dispensing data are a good proxy for actual usage. In addition, prescribing itself as a process indicator carries an important meaning, as it is the first step to accessing benzodiazepines and related drugs. Third, although the OECD guidelines’ suggested indicators are only measured for prescribing undertaken in primary care, we also included prescribing by hospitals, which made it difficult to compare our findings to international data. However, our inclusion criteria are more likely to reflect the Korean situation, because the Korean Government allows hospitals to provide a large scale of services to outpatients, and patients can choose any clinic or hospital without a referral slip [31]. Lastly, the inappropriate prescription of benzodiazepines was measured only in terms of a quantitative approach, based on long-term use. In spite of concerns whether this aspect or some other dimensions really capture the inappropriate prescribing of benzodiazepines, the OECD’s PQIs have been agreed upon by a range of stakeholders to be a valid and useful method to measure or monitor the prescribing quality at the population-level. [9]

## Conclusions

A small decreasing tendency was observed in the prescribing of long-acting benzodiazepines, some of the most commonly prescribed drugs, but with a high potential for inappropriate use, in elderly patients in Korea. Although limited information was available about the prescribing physician, the inappropriate long-term use of benzodiazepines in the elderly was significantly related to the patients and physicians’ behaviors, as well as the patients’ demographic and clinical characteristics. Introducing prescribing quality indicators (PQIs) in primary health care may be useful for lowering the prescription rate of potentially inappropriate drugs. We suggest that policy efforts, such as periodic assessment of prescribing, restricting prescribing days, and more practical guidelines, are needed to improve the prescribing quality.

## Abbreviations

ATC: Anatomical-Therapeutic-Chemical
DDD: Defined Daily Dose
DUR: Drug utilization review
HCQI: Health Care Quality Indicator
HIRA: Health Insurance Review & Assessment Service
OECD: Organization for Economic Cooperation and Development
PQI: Prescribing quality indicators
